# Improved cancer detection in Waldeyer’s tonsillar ring by ^68^Ga-FAPI PET/CT imaging

**DOI:** 10.1007/s00259-020-05055-8

**Published:** 2020-10-15

**Authors:** S. Serfling, Y. Zhi, A. Schirbel, T. Lindner, T. Meyer, E. Gerhard-Hartmann, C. Lapa, R. Hagen, S. Hackenberg, A. K. Buck, A. Scherzad

**Affiliations:** 1 Department of Nuclear Medicine, University Hospital Wuerzburg, Oberduerrbacher Str. 6, 97080 Wuerzburg, Germany; 2 Department of Otorhinolaryngology, Plastic, Aesthetic and Reconstructive Head and Neck Surgery, Julius Maximilian University of Wuerzburg, 97080 Wuerzburg, Germany; 3 Department of Nuclear Medicine, University Hospital Heidelberg, 69120 Heidelberg, Germany; 4 Department of Pathology and Comprehensive Cancer Center Mainfranken, Julius Maximilian University of Wuerzburg, 97080 Wuerzburg, Germany; 5 Department of Nuclear Medicine, University Hospital Augsburg, 86156 Augsburg, Germany

**Keywords:** Waldeyer’s tonsillar ring, Cancer of unknown primary (CUP), Positron emission tomography/computed tomography (PET/CT)

## Abstract

**Purpose:**

In cancer of unknown primary (CUP), positron emission tomography/computed tomography (PET/CT) with the glucose analog [^18^F]FDG represents the standard imaging approach for localization of the malignant primary. Frequently, however, [^18^F]FDG PET/CT cannot precisely distinguish between small occult tumors and chronic inflammation, especially in Waldeyer’s tonsillar ring. To improve the accuracy for detecting primary tumors in the Waldeyer’s tonsillar ring, the novel PET tracer [^68^Ga]Ga-FAPI-4 for specific imaging of fibroblast activation protein (FAP) expression was used as a more specific target for cancer imaging.

**Methods:**

Eight patients with suspicion of a malignant tumor in Waldeyer’s tonsillar ring or a CUP syndrome were examined. PET/CT scans with [^18^F]-FDG and [^68^Ga]Ga-FAPI-4 were performed for pre-operative tumor localization. After surgical resection, histopathological and immunohistochemical results were compared to PET/CT findings.

**Results:**

Histopathology revealed a palatine or lingual tonsil carcinoma in all patients. In case of lymph node metastases smaller than 7 mm in size, the [^18^F]FDG PET/CT detection rate of cervical lymph node metastases was higher than that of [^68^Ga]FAPI PET/CT, while both tracers identified the primary tumors in all eight cases. The size of the primary and the lymph node metastases was directly correlated to the respective FAP expression, as detected by immunohistochemistry. The mean SUV_max_ for the primary tumors was 21.29 ± 7.97 for ^18^F-FDG and 16.06 ± 6.29 for ^68^Ga-FAPI, respectively (*p* = 0.2). The mean SUV_max_ for the healthy contralateral tonsils was 8.38 ± 2.45 for [^18^F]FDG and 3.55 ± 0.47 for [^68^Ga]FAPI (*p* < 0.001). The SUV_max_ ratio of [^68^Ga]FAPI was significantly different from [^18^F] FDG (*p* = 0.03). Mean TBR_max_ for the [^68^Ga]Ga-FAPI-4 tracer was markedly higher in comparison to [^18^F]FDG (10.90 vs. 4.11).

**Conclusion:**

Non-invasive imaging of FAP expression by [^68^Ga]FAPI PET/CT resulted in a better visual detection of the malignant primary in CUP, as compared to [^18^F]FDG imaging. However, the detection rate of lymph node metastases was inferior, presumably due to low FAP expression in small metastases. Nevertheless, by offering a detection method for primary tumors with the potential of lower false positive rates and thus avoiding biopsies, patients with CUP syndrome may benefit from [^68^Ga]FAPI PET/CT imaging.

## Introduction

The incidence of head and neck squamous cell carcinoma (HNSCC) is more than 600,000 new cases per year worldwide, while almost half of the patients die from this disease [1]. The progression of the disease as well as the individual prognosis is influenced by several factors, including tumor size and localization, extranodal tumor spread, presence of distant metastases, and the degree of tumor differentiation [2]. With a percentage of 2–9% HNSCC manifests itself in clinical practice only through cervical lymph node metastases, while no obvious evidence of a primary tumor is observed [3]. This subgroup is defined as cancer of unknown primary (CUP), which requires a detailed medical history. Clinical examination and pre-operative imaging are important to localize the malignant primary. Using computed tomography (CT), the detection rate of the primary tumor in CUP is approximately 16% or up to 41% combining information from CT and magnetic resonance imaging [4]. Positron emission tomography/computed tomography (PET/CT) with fluorodeoxyglucose ([^18^F]FDG) has been established in Germany as gold standard for primary pre-operative staging in CUP [5]. The detection rate of the primary tumor was improved to 86%, with a specificity of 69% by using [^18^F]FDG PET/CT (FDG PET/CT) [6, 7].

In most patients with CUP, occult primary tumors are localized in the palatine tonsils and the base of the tongue [8]. However, tumors in the Waldeyer’s tonsillar ring are difficult to distinguish from chronic inflammation in FDG PET/CT scans. This leads to frequent false positive findings which occur in up to 39% of cases [9]. Due to a lack of accuracy, panendoscopy accompanied by multiple biopsies, bilateral tonsillectomy, and superficial tongue base laser resection is necessary [10]. However, these invasive procedures are associated with postoperative risks, such as postoperative bleeding. A higher specificity of diagnostic imaging would allow a better identification rate of small oropharyngeal cancers which—due to artifacts—are not detectable by FDG PET/CT. In those cases, diagnostic tonsillectomy could be avoided.

Cancer-associated fibroblasts (CAFs) are located within the tumor stroma of most epithelial tumors and belong to a subpopulation of fibroblasts [11]. By secreting various cytokines, chemokines and growth factors CAFs play an essential role for the tumor microenvironment. Often the invasiveness of the tumor invasion is promoted by the interaction between CAFs and tumor cells, for instance in oral squamous cell carcinoma [12]. A hallmark of CAFs is the overexpression of fibroblast activation protein (FAP) on their cell surface [13]. FAP corresponds to a type II transmembrane glycoprotein that acts as a serine protease of the DiPeptidyl-peptidase family (DPP) [11]. Expression of FAP was described in association to diverse carcinomas, such as in breast and colorectal carcinomas, and in oropharyngeal cancer [11, 14, 15]. In its active dimeric form, FAP acts as an exo- and endopeptidase, influencing the proliferation, migration, and invasion of tumor cells [15–18]. In contrast, FAP is absent in normal adult tissues [19].

The radiolabeled FAP-targeted inhibitor [^68^Ga]Ga-FAPI-4 (FAPI) was developed as a tracer for PET/CT imaging [20]. The aim of this study was to improve the detection rate and localization of suspected palatine and lingual tonsil carcinomas by non-invasive imaging. For this reason, we compared this novel tracer with the standard [^18^F]FDG imaging.

## Patients, materials, and methods

### Patients

In the current retrospective study, we analyzed eight patients who were treated for suspected carcinomas of the Waldeyer’s tonsillar ring from November 2018 to January 2020. Initially, the suspected diagnosis was an oropharyngeal cancer or rather a CUP syndrome as differential diagnosis. All patients received FDG PET/CT as pre-operative staging for tumor detection. In order to distinguish tumor tissue more precisely from inflammatory tissue, an additional FAPI PET/CT scan was performed. The results were compared to a control group (*n* = 15) that had no oral cancer (Table 1). [^68^Ga]Ga-FAPI-4 was offered on a compassionate use base and in compliance with “37 of Declaration of Helsinki and the German Medicinal Products Act, AMG” 13 2b. All patients were informed and gave their written consent for pre-operative imaging. Subsequently, a diagnostic panendoscopy was carried out with biopsy of the suspected lesion. The TNM staging system was applied according to the American Joint Committee on Cancer (AJCC) classification of head and neck cancer. After the histopathological analysis confirmed the primary tumor, all patients received tumor resection and neck dissection of the affected side. The immunohistochemical stainings for FAP in the resected tissues—in comparison to PET/CT imaging—were approved by the Ethic Committee of the Medical Faculty, University of Wuerzburg (no. 123/19).

**Table 1:** Control group (*n* = 15) with a mean age of 60 years (range 43–81; male 67%, female 33%)

Control group	SUV_max_ right tonsil	SUV_max_ left tonsil	SUV_max_ right tonsil	SUV_max_ left tonsil
1	10.37	9.36	2.65	2.63
2	3.12	3.59	1.47	1.42
3	8.48	7.63	2.82	3.81
4	3.45	3.85	3.03	3.41
5	7.44	5.64	3.67	3.27
6	6.32	5.95	2.51	2.12
7	3.91	3.91	3.67	3.52
8	6.34	6.05	3.57	2.79
9	6.44	6.51	3.63	3.66
10	5.95	7.96	4.32	4.24
11	5.79	6.93	3.84	3.08
12	7.13	4.93	2.67	2.88
13	12.32	9.69	4.01	3.97
14	7.31	4.91	2.61	1.89
15	5.06	5.62	2.65	2.36
**Mean SUV**_**max**_	**6.63**	**6.17**	**3.14**	**3.04**
**SUV**_**max**_ **ratio**	**1.18**		**1.11**	

### Patient data

The mean patient age was 62 years (with a range of 58–72 years). In all cases, tumors were transoral resected and immediately checked by an experienced pathologist with regard to the resection margins. In case of a positive resection margin, a further resection in combination with a neck dissection of the tumor-affected side was performed. Subsequently, a detailed analysis of the tumor with regard to grading, perineural infiltration, or HPV infection status was performed. The lymph nodes, which were resected in the course of neck dissection, were fixed in formalin. Subsequently, the presence of metastases was analyzed by an experienced pathologist.

**Fig. 1:**
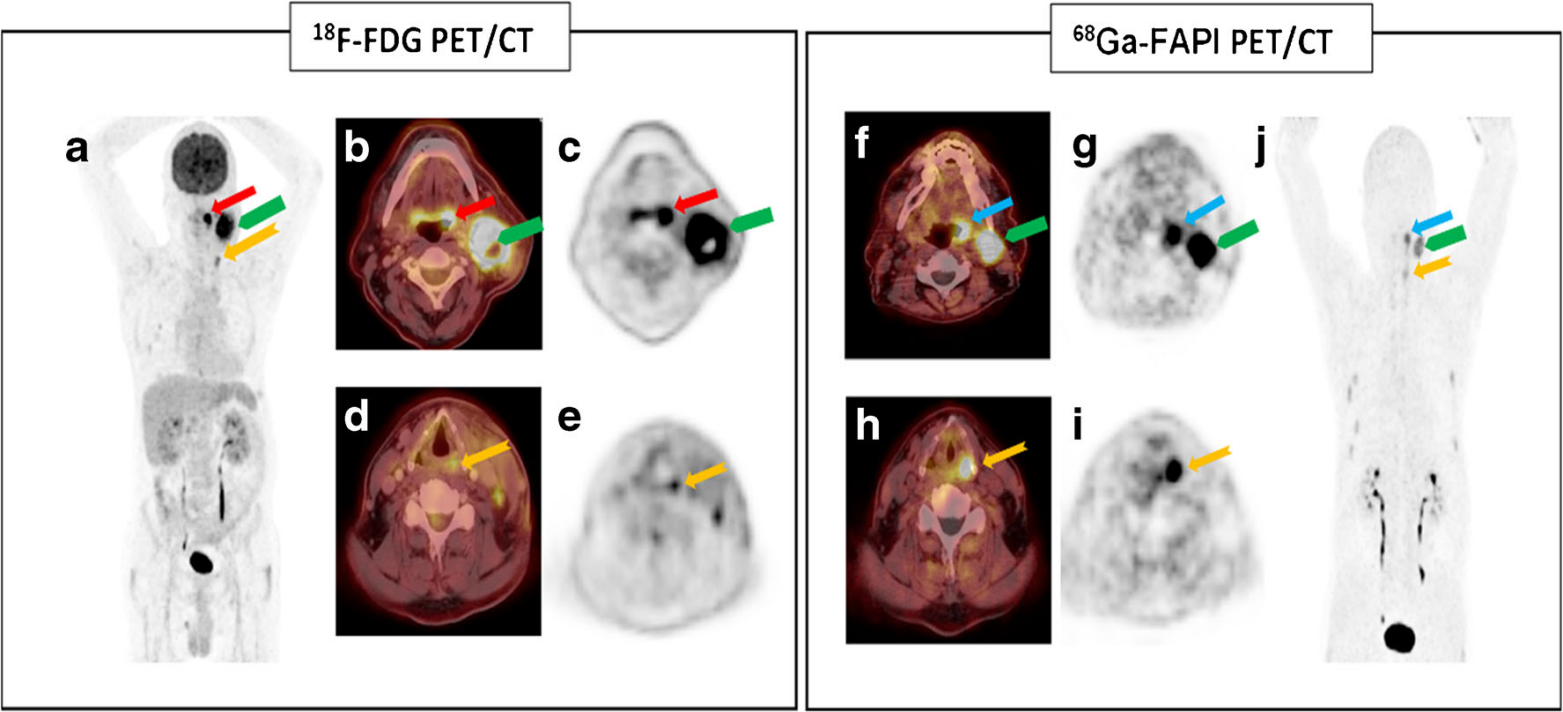
PET/CT scans with the FDG and FAPI tracer of patient with palatine tonsil carcinoma and simultaneous hypopharyngeal carcinoma. **a**, **b**, **c**, **d**, **e** Coronar and axial slices of a partial body scan of FDG PET/CT from patient #4. All five sections show a strong tracer uptake in the left palatine tonsil (red arrow), but also in the contralateral normal tonsil. The ipsilateral neck lymph node metastasis with an intensive tracer uptake is marked by a green arrow. As an incidental finding, a second carcinoma of hypopharynx was detected (yellow arrow). **f**, **g**, **h**, **i**, **j** Coronar and axial sections of a partial body scan of FAPI PET/CT from the same patient. In FAPI PET-CT, the primary tumor (blue arrow), the cervical lymph node metastasis (green arrow), and the hypopharynx carcinoma (yellow arrow) show an intensive tracer uptake. The contralateral normal tonsil shows a very poor tracer uptake

All patients showed a histopathological result of squamous cell carcinoma. Seventy-five percent of which were positive for p16/HPV (human papillomavirus)-positive (Table 2). One patient (patient #4) had a p16-negative T3 tonsil carcinoma and a simultaneous T1 hypopharyngeal carcinoma (Fig. 1). The second carcinoma was removed by transoral laser microsurgery in the same surgical session. In two patients (patients #2 and #3), an additional tracer uptake in the lung was detected by PET/CT during pre-operative staging. In patient #2, histopathological examination revealed a primary lung carcinoma as secondary malignancy. In patient #3, histopathological work-up of the lung lesion also revealed a squamous cell carcinoma, whereas the distinction between primary lung carcinoma and a metastatic lesion was impossible based on histopathology. Six out of 8 patients (75%) received adjuvant radiochemotherapy with a radiation dose of up to 66 Gy and 4 cycles of cisplatin.

**Table 2:** Patient characteristics (age, sex, and smoker) and the tumor characteristics (localization and p16/HPV-status)

Patient	Age	Sex	Smoker	Tumor localization	p16-status
1	58	F	No	Palatine tonsil right side	Positive
2	72	M	Yes	Palatine tonsil right side	Negative
3	54	M	No	Lingual tonsil right side	Positive
4	61	M	Yes	Palatine tonsil left side	Negative
5	61	M	No	Palatine tonsil right side	Positive
6	68	F	Yes	Palatine tonsil left side	Positive
7	59	M	Yes	Palatine tonsil left side	Positive
8	62	M	Yes	Palatine tonsil right side	Positive

At the last time point of clinical follow-up (March 2020), six out of 8 patients (75%) were in complete remission. Only patient #4 showed a progressive disease with new pulmonary metastases during restaging with FDG PET/CT in May 2019, which were not detected pre-operatively. Patient #6 was lost to follow-up.

### Imaging and image analysis

All pre-operative PET/CT examinations were performed on a Siemens Biograph mCT 64, (Siemens Healthineers, Erlangen, Germany). The investigation was carried out after the initial presentation of the patient at the Dept. of Otolaryngology, Head and Neck Surgery of the University Hospital Wuerzburg. Before imaging, the blood glucose levels were tested in all patients and the metrics were below 165 mg/dl in all patients. Sixty minutes after intravenous injection of approximately 300 MBq ^18^F-FDG (average 292 ± 32 MBq), imaging was performed. First, a full-body protocol with lifted arms was performed, followed by a head and neck protocol (i.e., arms in down-position). In the head and neck protocol, the patients were imaged from the base of the skull to the upper thoracic aperture with a 30-cm field of view and a 128 × 128 matrix. PET section thickness was 5 mm. All PET images were reconstructed iteratively.

Within 4 to 7 days, FAPI PET/CT was performed in low dose technique. One hundred fifty megabecquerels (average 145 MBq) of [^68^Ga]Ga-FAPI-4 were injected intravenously. After a 60-min incubation time, the abovementioned full body as well as the head and neck protocols were performed, as mentioned above. Data evaluation was carried out using the manufacturer’s software (syngo MI.PET/CT; Siemens Healthineers, Erlangen, Germany). PET/CT image interpretation was based on visual and semiquantitative analysis and evaluated by two experienced readers. Visual identification of a malignant tumor in Waldeyer’s tonsillar ring was obtained by the asymmetry of high tracer uptake in one of both tonsils. Caused by inflammatory changes, the tracer [^18^F]FDG (FDG) has a strong physiological uptake in both tonsils (range SUV_max_ 2.1–11.4) [21]. The physiological SUVmax ratio for FDG is between 0.70 and 1.36 in palatine tonsils [22]. For semiquantitative analysis, a SUV_max_ ratio between tonsils of ≥ 1.6 for ^18^F-FDG [21] and ≥ 1.5 for FAPI—in comparison to the control group (Table 1)—were defined as PET positive for malignancy. Only lymph nodes with high focal uptake (SUV_max_ ≥ 4.0 for FDG; SUV_max_ ≥ 3.0 for FAPI) were classified as suspicious for malignancy. Consequently, lower focal FDG and FAPI uptake with matching node-like structures at CT scans were considered as abnormal, irrespective of lymph nodal size.

The tumor suspected area in the pharyngeal was derived from the region with the highest tracer uptake in correlation to the clinical data. For the further analysis of normal tissue and tumor tissues, regions of interest (ROIs) were defined with a fixed diameter of 10 mm. The maximum standardized uptake value (SUV_max_) was calculated within the ROI as the most important comparative value of the two tracers using the manufacturer’s algorithm. The SUV_max_ of the contralateral tonsil was calculated in the same way by defining the location with the most intensive tracer uptake. The background SUV_max_ was calculated from the aorta descendens. The ratio of SUV_max_ values (SUV_max_ ratio) has been defined at the quotient of the SUV_max_ of primary tumor and the normal contralateral tonsil. This value is a quantitative measure to express the asymmetrical tracer uptake in carcinomas and normal tonsils. The tumor-to-background ratio (TBR) is defined as quotient SUV_max_ of the tonsil carcinoma divided by the background SUV_mean_.

### Immunohistochemistry

Immunohistochemistry for FAP was performed according to standard immunohistochemical protocols on formalin-fixed paraffin-embedded tissue slides using an antibody generated against the fibroblast activation protein alpha (Abcam, ab207178; dilution 1:250). The immunostaining was assessed by a scoring system adopted from Henry et al. [23]. The staining of stromal cells adjacent to the carcinoma infiltrate was assessed as 0 (absence of FAP immunostaining), 1+ (weak staining in < 10% of the tumor stromal cells), 2+ (positive FAP immunostaining in 10% to 50% of surrounding stromal cells), and 3+ (moderate to strong FAP immunostaining in > 50% of surrounding stromal cells). Tumor-free lymph nodes showing negative staining for FAP together with tumor-free tonsils were used as negative control.

### Statistical analysis

The statistical evaluation was performed using Microsoft Excel (Version 16.17). For descriptive statistics, the quantitative values were given as mean value ± standard deviation. Two-sample *t* tests were performed to compare FAPI and FDG metric measurements. A *p* value of 0.05 indicated statistical significance. A *p* value of 0.001 indicated highest statistical significance.

## Results

### Imaging results of primary tumors

In this study, the established tracer FDG was compared to the FAPI tracer with regard to tracer uptake in cancer of Waldeyer’s tonsillar ring and correlated to the tumor size and FAP expression.

**Fig. 2:**
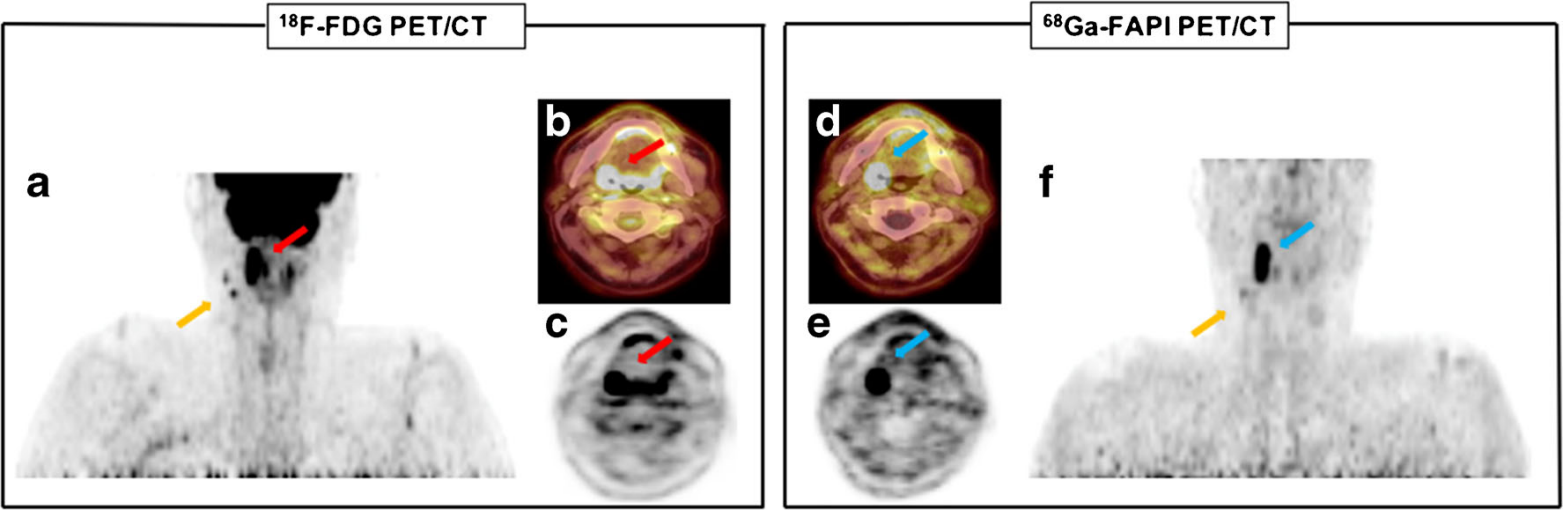
PET/CT scans with the FDG and FAPI tracer of patient with palatine tonsil carcinoma. **a**, **b**, **c** Coronar and axial slices of a partial body scan by FDG PET/CT in patient #2. An intensive tracer uptake was detected in palatine tonsils of both sides. The red arrow marks the carcinoma of the right side, the orange arrow marks two lymph node metastases **d**, **e**, **f** Coronar and axial slices of a partial body scan by FAPI PET/CT from the same patient. Due to the intensive tracer uptake of FAPI (blue arrow), in contrast to the FDG PET/CT, the right palatine tonsil can be clearly identified as carcinoma. The left palatine tonsil shows a very low tracer uptake. Only one of two lymph node metastasis were seen on FAPI PET/CT (orange arrow)

**Fig. 3:**
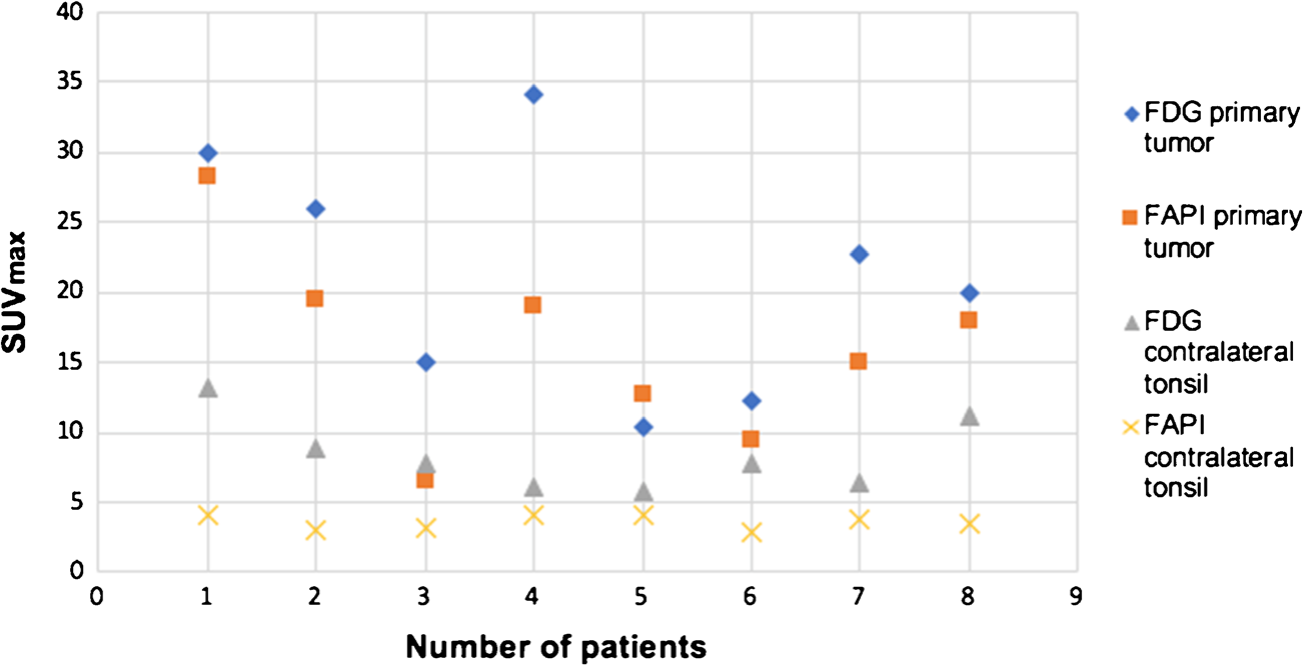
Compilation of FDG and FAPI tracer uptake in primary tumor and contralateral healthy tonsils. In contrast to FDG tracer, the contralateral healthy tonsils showed a very low FAPI tracer uptake (***p < 0.001)

All primary tumors were detected by both tracers while FDG showed higher uptake in all cases. Compared to contralateral normal tonsils the mean SUV_max_ of the tumor tissue was significantly higher in FDG imaging (*p* = 0.003) and even more significant for FAPI PET/CT (*p* = 0.001; Fig. 2). In addition, all normal tissues of the Waldeyer’s tonsillar ring showed a very low tracer uptake in FAPI PET/CT scans (Table 3). The uptake of FAPI in the normal tonsils was significantly lower than that of FDG (*p* < 0.001) (Table 4 and Fig. 3). The mean SUV_max_ of the control group for the right healthy tonsil was 6.6 for FDG and 3.2 for FAPI, as well as 6.2 (FDG) and 3.0 (^68^FAPI) for the left healthy tonsil. The mean SUV_max_ values of both tracers in the tonsil carcinomas was markedly higher than surrounding tissue with 21.3 ± 10.0 (FDG) and 15.9 ± 6.3 (FAPI). When comparing mean SUV_max_ for both tracers of the contralateral healthy tonsil, a significant difference of 8.2 ± 2.7 for FDG and 3.4 ± 0.58 for FAPI was observed. Consequently, the SUV_max_ ratio of FDG was only 2.68 (range 1.56–5.59) and 4.10 (range 2.13–6.88) in case of FAPI. Moreover, FDG TBR_max_ mean was less than a half with 4.11 (range 2.5–7.1) compared to FAPI with a value of 10.89 (range 6.3–27.5) (Table 4).

**Table 3:** SUV_max_/SUV_max_ ratio measurement of palatine and lingual tonsils. SUV_max_ mean and SUV_max_ ratio mean. The SUVmax mean significantly different between primary tumor and contralateral healthy tonsil in FDG PET/CT (**p = 0.003) and FAPI PET/CT (***p = 0.001). Also the SUV_max_ ratio of FAPI tracer distinguishes significantly from SUV_max_ ratio of FDG tracer (*p = 0.03)

	Primary tumor	Contralateral tonsil
FDG group (*n* = 8)		
SUV_max_ mean	21.29**	8.38
Standard deviation	7.97	2.45
Range	10.35–34.1	5.8–13.2
SUV_max_ ratio mean	2.68	
Standard deviation	1.27	
Range	1.56–5.59	
FAPI group (*n* = 8)		
SUV_max_ mean	16.06***	3.55
Standarddeviation	6.29	0.47
Range	6.6–28.2	2.9–4.1
SUV_max_ ratio mean	4.47*	
Standard deviation	1.55	
Range	2.13–6.88

**Table 4:** Tumor characteristics and imaging results. Data from all eight patients itemized according to T-stage and size of the primary tumor in palatine or lingual tonsil, SUV_max_, SUV_max_ ratio of both tracers in primary tumor and contralateral healthy tonsil and TBR_max_ of both tracers

Patient no.	Tumor extension	FDG SUV_max_ primary tumor	FDG SUV_max_ contralateral tonsil	FDG SUV_max_ ratio	FDG TBR_max_	FAPI SUV_max_ primary tumor	FAPI SUV_max_ contralateral tonsil	FAPI SUV_max_ ratio	FAPI TBR_max_
1	T2 21 × 17 mm	30	13.2	2.27	4	28.2	4.1	6.88	27.5
2	T2 35 × 13 mm	26	8.8	2.95	5.2	19.5	3	6.5	12.1
3	T3 40 × 9 mm	15	7.7	1.95	2.6	6.6	3.1	2.13	3.22
4	T3 47 × 8 mm	34.1	6.1	5.59	7.1	19	4.1	4.63	10.8
5	T1 11 × 8 mm	10.35	5.8	1.78	2.6	12.7	4	3.18	7
6	T2 22 × 8 mm	12.2	7.8	1.56	2.5	9.5	2.9	3.28	6.3
7	T2 22 × 15 mm	22.7	6.4	3.55	6	15	3.7	4.05	10.7
8	T3 41 × 9 mm	20	11.2	1.79	2.9	18	3.5	5.14	9.5

### Imaging results of lymph node metastases

All patients received a neck dissection as part of their therapy. A total of 173 lymph nodes were dissected and histopathologically analyzed. The results of the analyses were compared to the PET/CT imaging findings. A total of 17 lymph node metastases could be confirmed histopathologically (Table 5). Histopathological results were compared to the findings at the FDG PET/CT scan. Fourteen out of 17 lymph node metastases showed an intensive pre-operative uptake of FDG. In contrast, only 8 lymph node metastases showed an intensive uptake in FAPI scans. Furthermore, histopathological results indicated that 156 out of 173 resected lymph nodes were tumor-free, and 3 lymph nodes described as hypermetabolic upon FDG PET/CT imaging were false positive. In contrast, none of the lymph nodes showed a false positive result by FAPI PET/CT imaging, as detected upon operation. Table 5 compiled for each patient the number and size of histopathological positive and PET positive cervical lymph nodes and the FAP expression.

**Table 5:** Resected primary tumor and cervical lymph node metastases in correlation with PET/CT scans and immunostaining. TNM-status of the tumor, HPV (+/-), amount of lymph node metastases, the lymph node metastases subdivision in FDG positive and FAPI positive lymph nodes, FAP expression for each lymph node metastases and primary tumor, primary tumor size

Patient	TNM	HPV (+/-)	Lymph node metastases size (mm)	FAPI/FDG (+/-)	Immunostaining (0–3)		Primary tumor size (mm)
1	T2N1M0	+	4	-/-	1+	3+	21 × 17
			6	-/+	2+		
			7	-/+	1+		
			29	+/+	3+		
2	T2N3bM0	-	7	-/+	3+	3+	35 × 13
			7	+/+	2+		
3	T3N1Mx	+	62	+/+	3+	3+	40 × 9
4	T3N3bM0	-	4	-/+	1+	3+	47 × 8
			11	+/+	2+		
			12	+/+	3+		
5	T1N1M0	+	27	+/+	3+	2+	11 × 8
6	T2N2bM0	+	32	+/+	3+	2+	22 × 8
			6	-/-	1+		
			11	-/-	0		
7	T2N1M0	+	50	+/+	3+	2+	22 × 15
8	T3N1M0	+	4	-/+	1+	3+	41 × 9
			9	+/+	3+		

### Imaging results of secondary primaries

In two patients, concomitant secondary malignancies in the lung were detected. Both tracers detected a secondary primary of the lung in patient #2 (histological confirmed squamous cell carcinoma pT1b; 12 mm × 13 mm; FDG: SUV_max_ 12.83; FAPI: SUV_max_ 6.26). In patient #3, it was impossible to distinguish between a primary lung carcinoma or a metastasis. Interestingly, only FDG showed an uptake in this 8-mm malignancy with a SUV_max_ of 3.91 (SUV_max_ 0.59 for FAPI).

### Correlation of imaging results with immunohistochemistry

**Fig. 4:**
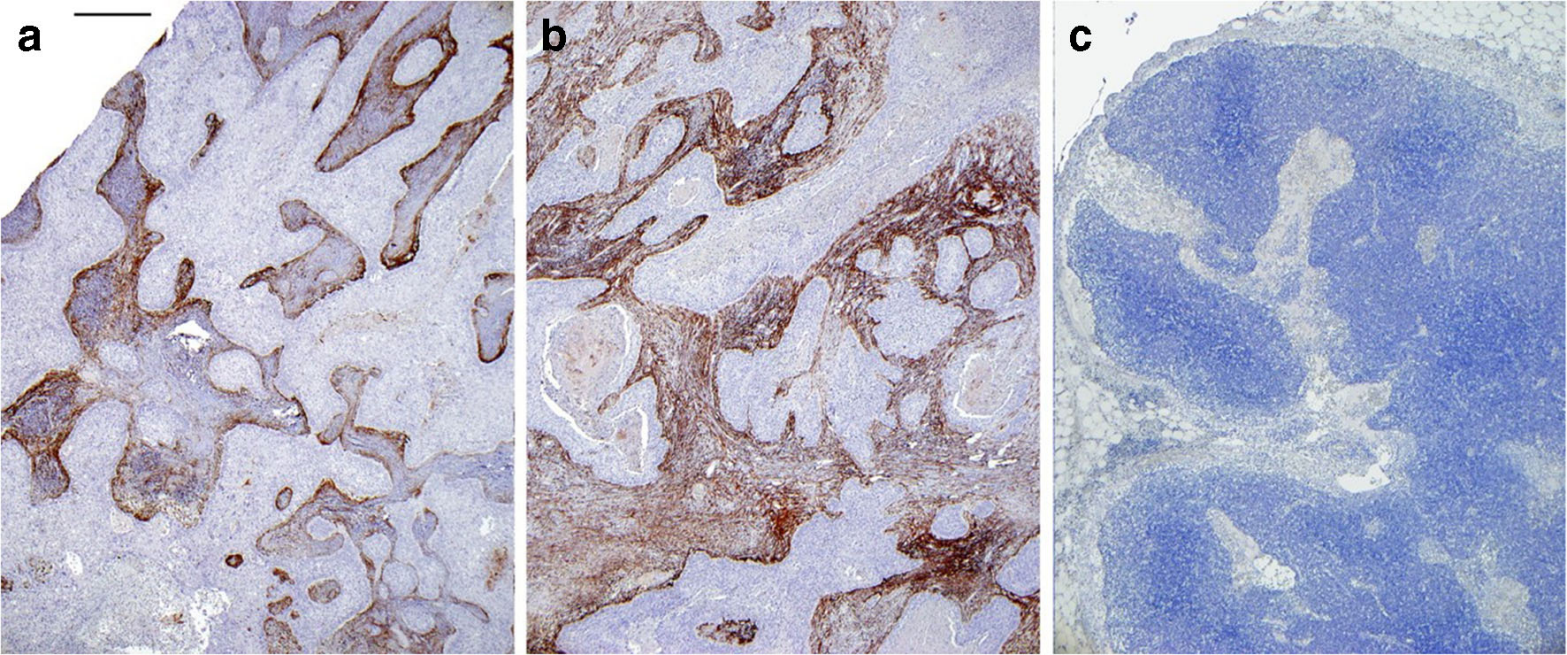
Immunohistochemistry of resected tissue from patient #4. FAP alpha immunohistochemistry from patient #4 (with positive staining of stromal cells adjacent to the carcinoma infiltrate of the primary tumor (**a** length of scale bar 500 μm) and a **b** lymph node metastasis. **c** No staining for FAP alpha was observed in a tumor free lymph node

With the immunohistochemical staining with the antibody, which is specific for the FAP alpha protein, all primary tumors and all lymph node metastasis except one were positive for FAP alpha. The largest primary tumors (6 of 8) showed a score of 3+ immunostaining for FAP, and one T1 and a small T2 primary tumor were scored 2+. In lymph node metastases of the size between 4 and 6 mm, there was only a weak FAP expression (score 1+) without FAPI uptake. The score 2+ had 6- to 11-mm large lymph node metastases which were detected by FAPI PET/CT. With a size of 12 mm, all lymph node metastases (score 3+) were detected by the FAPI tracer. Only one lymph node metastasis had a score of 3+ with the size of 7 mm. The lymph node metastases (11 mm size) with no FAP expression (score 0) remained undetected by both tracers. Figure 4 gives an example of the FAP alpha protein expression by immunohistochemistry in the detected primary tumor and a lymph node metastasis of patient #4. The exact FAP expression for each lymph node metastases are shown in Table 5.

## Discussion

Initial staging is a fundamental element in diagnosis of head and neck squamous cell carcinoma (HNSCC) and has major impact on tumor therapy [24]. The most common location of the occult primary tumors from clinical CUP syndrome are the tonsils and the base of the tongue [25]. In comparison to conventional imaging, such as CT or MRI, FDG PET offers a higher sensitivity and specificity for tumor detection [26]. After phosphorylation of FDG, tumor cells with increased anaerobic glycolysis are predominantly labeled by the intracellular accumulation of FDG tracer (metabolic trapping) [27]. However, the specificity of FDG is limited and frequently leads to false positive results, in particular due to inflammatory tissues [25, 28]. In those cases, panendoscopy including bilateral tonsillectomy and biopsies of tongue base and nasopharynx are part of the procedure to detect primary tumors. But the risk of a secondary bleeding after tonsillectomy can occur in up to 5% of treated patients and can have fatal consequences for the patients [29].

In contrast to the metabolism targeted tracer FDG, FAPI reacts specifically with the surface protein FAP on CAFs which can be found in tumor stroma of many squamous cell carcinomas [11]. In this study, both tracers (FDG and FAPI) were used for the detection of carcinomas in Waldeyer’s tonsillar ring. Asymmetrical uptake of both radiotracers was found in all patients with preference in the tumor affected side (FDG mean SUV_max_ 21.29; FAPI mean SUV_max_ 16.06). In addition, the SUV_max_ of the non-malignant contralateral tonsil was significantly lower for FAPI (3.55 ± 0.47) compared to FDG PET/CT imaging (8.38 ± 2.45) (*p* < 0.001). The corresponding SUV_max_ ratio for the FAPI tracer was higher in almost all patients and differed significantly from the FDG tracer. The distinct differences in the SUV_max_ ratio between both tracers is based mainly on the very low uptake of FAPI in the contralateral healthy tonsil in all patients, compared to the tumor affected side (*p* = 0.001). In addition, the mean TBR_max_ of the FAPI tracer was much higher in comparison to the FDG tracer (10.90 vs. 4.11). Due to the higher mean values of TBR_max_ and SUV_max_, it is much easier to visually detect tumor tissue and to separate it from inflammatory changes. All primary tumors showed a correlation of size and intensity of FAP immunostaining. One T1 and one small T2 primary tumor showed a moderate FAP expression (score 2+), the larger tumors (T2 and T3) had a high FAP expression of surrounding stromal cells (score 3+). Hence, false positive results in tonsils and inflammatory tissues could be avoided. This benefit of FAPI PET/CT imaging may render biopsies in nasopharynx unnecessary for CUP patients as well as bilateral tonsillectomy unnecessary for CUP patients. Therefore, tracer uptake in the primary tumor could easily be differentiated from the physiological uptake of surrounding tissue by FAPI PET/CT imaging. Consequently, small tumors of T1 stage could be detected more effectively by FAPI PET/CT imaging, and false negative results might be reduced.

Apart from the detection of the primary tumor which is essential for the planning of resective surgery, imaging modalities are a key element for the diagnosis of cervical lymph node metastases and distant metastases. For the detection of cervical lymph node metastases, FDG PET showed a higher sensitivity than CT. Our study confirmed previous findings by Yamazaki et al. [30]. In this study, it was reported that lymph node metastases below 5 mm are usually not recognized by FDG PET. With a lymph node metastases size of 5 to 9 mm, the detection rate was 45%, and it rose to almost 100% by a lymph node metastases size more than 10 mm [27]. In our study, mainly HPV-positive carcinomas of palatine and lingual tonsil with cervical lymph node metastases (75%) were involved. By FAPI PET/CT imaging, the detection rate of cervical lymph node metastases was independent from HPV association. The detection rate of lymph node metastases was 47% in FAPI PET/CT (vs. 82% in FDG PET/CT). One reason could be a reorganization process of the tumor microenvironment, which is reported to be induced by HPV-positive epithelia. These structural changes include thinning of the basal membrane, apparent degradation and disruption of the collagen fibril network, and additional disintegration of the extracellular matrix [31]. Thus, the lower sensitivity for small metastases could be explained by a delayed conversion of normal fibroblasts towards FAP overexpressing CAFs [32]. This would result in a lower FAP occurrence in cervical lymph node metastases especially in early stages of metastasis, which was also apparent in immunohistochemistry.

The FAP expression of lymph node metastases also appears to correlate with the size of the lesion. Lymph nodes up to 7 mm in size showed only a weak FAP expression in less than 10% of the surrounding tumor-associated stromal cells (stain score 1+). This resulted in a negative FAPI scan. In contrast, all lymph node metastases between 7 and 62 mm in size were detected pre-operatively by using FAPI and FDG (stain score 2+/3+). Probably due to its cystic morphology, a sole lymph node metastasis with a size of 11 mm showed a negative FAP immunostaining and no tracer uptake in imaging. The timespan of tumor development of cervical lymph node metastases is certainly an important parameter for the transformation of normal fibroblasts into CAFs, and the overexpression of FAP as a target for the FAPI tracer.

There are several limitations of our study. These include the very small number of patients and the unbalanced distribution of HPV-positive and -negative tumors. Furthermore, due to the lack of follow-up, analysis of the long-term prognosis analysis regarding disease-free and overall survival is currently not feasible. In the future, a larger patient cohort will be necessary to study FAPI PET/CT in HNSCC. Especially immunohistochemical studies of HPV-positive and -negative tumors are necessary to further investigate the interaction between primary tumor/cervical lymph node metastases and CAFs as well as FAP.

In conclusion, the differentiation between primary tumor and surrounding or contralateral normal tonsillar tissue is improved by FAPI as compared to the standard PET radiotracer FDG. Therefore, small occult cancers in the Waldeyer’s tonsillar ring may be detected by FAPI PET/CT with higher precision. Especially in patients with suspected CUP syndrome, FAPI PET/CT might reveal small primary tumors in the tonsils without the requirement of diagnostic bilateral tonsillectomy. A more precise therapy planning by means of improved PET/CT imaging, as well as the reduction of postoperative complications may lead to a better outcome of patients.
